# Molecular characteristics of norovirus in sporadic and outbreak cases of acute gastroenteritis and in sewage in Sichuan, China

**DOI:** 10.1186/s12985-022-01897-w

**Published:** 2022-11-08

**Authors:** Ranran Cao, Xiaozhen Ma, Ming Pan

**Affiliations:** Sichuan Center for Disease Control and Prevention, No.6 Zhong Xue Road, 610041 Chengdu, People’s Republic of China

**Keywords:** Norovirus, Acute gastroenteritis, Sporadic, Outbreak, Sewage, Molecular epidemiology

## Abstract

**Background:**

Norovirus is highly diverse and constant surveillance is essential for the prevention and control of norovirus gastroenteritis.

**Methods:**

From 2015 to 2019, fecal samples were collected from sporadic cases and outbreaks of acute gastroenteritis reported to Sichuan center for disease control and prevention. Sewage samples were collected from a wastewater treatment plant in Sichuan. All samples were tested for norovirus by real-time reverse transcription polymerase chain reaction. Norovirus-positive clinical samples were sequenced by Sanger sequencing. Sewage samples were sequenced by amplicon and virome sequencing.

**Results:**

A total of 1462 fecal samples were collected and 11 different norovirus genotypes were detected. GII.4 Sydney 2012[P31] and GII.3[P12] were the dominant genotypes in sporadic cases whereas GII.2[P16] and GII.17[P17] were the dominant genotypes in outbreaks. GII.3 was predominant in children 0–6 months of age during spring and summer, while GII.4 was predominant in children older than 6 months and in the autumn. The detection rate of GII.17[P17] increased with age. In sewage, 16 genotypes were detected. GII.3, GII.4, GI.1, and GI.2 were the dominant genotypes.

**Conclusion:**

This study demonstrated that multiple norovirus genotypes co-circulate in Sichuan. It is vital to continuously trace the genetic diversity of norovirus to give a future perspective on surveillance needs and guide vaccine design and policy decisions.

## Background

Norovirus is among the most important causes of acute gastroenteritis (AGE) worldwide [1]. While norovirus disease symptoms are usually mild and self-resolving, severe outcomes can occur in young children, the elderly and immunocompromised individuals [2]. Worldwide, approximately 90% of children have likely experienced at least one norovirus infection by the age of five. Each year, an estimated 70,000–200,000 children die as a result of norovirus infections, mostly in developing countries [3, 4]. In China, AGE due to norovirus has been classified as a Category C infectious disease according to the National Notifiable Diseases Reporting System [5]. In recent years, the etiology of 90% of AGE outbreaks in China has been linked to norovirus which poses a significant threat to public health [6].

Noroviruses are highly diverse due to frequent mutations and recombination. Currently at least 10 different norovirus genogroups are recognized [7]. Further, based on the complete amino acid sequences of VP1 gene and the partial nucleotide sequences of RNA-dependent RNA polymerase (RdRp) gene, noroviruses are classified into at least 48 genotypes and 60 polymerase types (P-types) according to a dual-typing method [7]. Multiple norovirus genotypes circulate worldwide and until 2012 new GII.4 variants emerged every two to four years, often resulting in an increased number of outbreaks. Constantly updating the molecular epidemiological data is essential for a comprehensive understanding of norovirus infections and for determining effective prevention measures. First, surveillance on molecular characterization could describe trends in genomic diversity of norovirus and predict the emergence of new variants. These data could further provide a future perspective on surveillance needs. Second, continuous changes in the diversity of noroviruses make it complicated to design a vaccine to induce full protection. Thus sustained surveillance on molecular characteristics is highlighted to assess possible immune escape and evolution by recombination so as to provide a full overview of norovirus epidemiology for future vaccine design and policy decisions. Third, molecular surveillance data can also provide evidence to trace the differences in norovirus genotypes among different regions, settings, age groups, susceptibility, mode of transmission and clinical manifestations. In view of the above, surveillance including molecular epidemiology is vital for control and prevention of norovirus-related gastroenteritis.

In 2016, CaliciNet China was established to monitor the molecular epidemiology of norovirus strains circulating in China. However, this network did not include data from norovirus studies conducted in Sichuan, a province in South western China. To increase the availability of molecular epidemiology data, this study analyzed sporadic and outbreak cases of AGE and environmental sewage to track the molecular characteristics of norovirus circulating in Sichuan. The goal was to provide baseline information on circulating norovirus genotypes for the development of future vaccines and interventions.

## Methods

### Sample collection

Fecal samples from sporadic cases were collected from children under five years of age who were hospitalized with AGE in a national viral AGE sentinel hospital in Sichuan from 2015 to 2019. According to the National Surveillance Protocol for Viral Diarrhea (2007 version), AGE was defined as three abnormal stools per day (liquid, watery, mucous, or bloody purulent) or less than three abnormal stools per day with vomiting.

Fecal samples from outbreak cases were collected from confirmed norovirus outbreaks in Sichuan from 2017 to 2019. According to the National Guidelines on Outbreak Investigation, Prevention and Control of Norovirus Infection (2015 version), a confirmed norovirus outbreak was defined at two levels: (1) the occurrence of five or more epidemiologically-linked AGE cases within three days with at least two norovirus-positive samples or (2) the occurrence of 20 or more epidemiologically-linked AGE cases within seven days with at least two norovirus-positive samples. All samples were collected three days after the onset of AGE and transported to viral diarrhea laboratory in Sichuan center for disease control and prevention (CDC) to be stored at − 80 °C.

Sewage samples were collected from a wastewater treatment plant in Sichuan that processes one million tons of wastewater daily. From July to December in 2019, one liter of wastewater was collected monthly from the inlets of the primary treatment tanks.

### Pre-treatment of samples

Fecal samples were suspended at 10% (W/V) with phosphate-buffered saline (Hyclone, United States), centrifuged at 5,000 g for 5 min, and the supernatant was collected. The sewage sample was concentrated by negatively-charged membrane filtration [8]. Briefly, the supernatant was collected after 2 h sedimentation at 4 °C followed by the addition of MgCl_2_ to the supernatant at a final concentration of 0.05 M. The pH was adjusted to 3.5–4.0 with 0.5 M HCl. The samples were then slowly passed through a negatively-charged membrane filter (Advantec, Japan) under gentle positive pressure. To elute the viruses, the filter with adsorbed viruses was cut into pieces and vortexed for 15 min in 15 mL of a 3% beef extract solution (pH 9.6) with glass beads. After centrifugation at 1,940 g for 30 min, approximately 10 mL eluents were collected, and the pH was adjusted to 7 with 0.5 M HCl. The final step was to pass the eluent through a 0.22 μm syringe filter to remove bacteria and fungi.

### RNA extraction

Viral RNA was extracted from 200 µL of the fecal sample supernatant and from 1000 µL of the sewage sample eluent using the Nucleic Acid Extraction System (Xi’an Tianlong Science & Technology, China) and following the manufacturer’s instructions.

### Real-time RT-PCR detection

All fecal and sewage samples were tested for norovirus by real-time reverse transcription polymerase chain reaction (RT-PCR) using a rotavirus/norovirus (GI and GII) nucleic acid testing kit (Mabsky, China) following the manufacturer’s instructions. Real-time RT-PCR was performed using the Applied Biosystems 7500 Real Time PCR System (Applied Biosystems, United States).

### Fecal sample sequencing

For norovirus-positive fecal samples, a one-step RT-PCR Kit (QIAGEN, United States) was used to amplify partial sequences of VP1 and RdRp genes. Primers MON432/G1SKR were used for GI and primers MON431/G2SKR were used for GII, yielding 579 bp and 570 bp RT-PCR products, respectively as described previously [9]. Subsequent Sanger dideoxy sequencing was carried out by Tsingke Biological Technology (Chengdu, China). The sequences obtained were assembled by Vector NTI Advance11.0. Edited sequences were analyzed and genotyped via an online Norovirus Typing tool (available at https://www.rivm.nl/mpf/typingtool/norovirus/). Nucleic acid similarities were analyzed by Bioedit 7.0.9.0.

### Sewage sample sequencing

Amplicon sequencing and virome sequencing were used to analyze the genome sequences of norovirus. Briefly, for amplicon sequencing, Superscript IV one-step RT-PCR system (Invitrogen, United States) was used to amplify partial sequences of the VP1 gene. Primers COG1F/G1SKR were used for GI and primers COG2F/G2SKR were used for GII, yielding 380 and 387 bp PCR products, respectively. The PCR products were used as a template in the second-round PCR using primers G1SKF/G1SKR for GI and G2SKF/G2SKR for GII, yielding 329 and 344 bp PCR products, respectively. Subsequent next generation sequencing was carried out by Tsingke Biological Technology (Chengdu, China) using an Illumina NovaSeq 6000 (PE150). After sequencing, raw reads were first filtered with Trimal v0.33. Clean reads were obtained after removing adapter sequences with Cut adapt v1.9.1. At the same time, Q20, Q30 and GC content of the clean reads were calculated. Subsequently, the paired-end reads were assembled with FLASH v1.2.11. The assembled clean reads were clustered to uniques.fa sequences with USEARCH v10.0 (identity threshold 1). Uniques.fa sequences were used for blast (blastn v2.09.0, E-value:1e-5) and for comparisons with database including all of the norovirus sequences detected in clinical samples in Sichuan and all the norovirus sequences could be down from NCBI (in-house norovirus database).

Virome sequencing was carried out by Novogene Co., Ltd. (Beijing, China). Briefly, the NEBNext® Ultra™ RNA Library Prep Kit was used to construct libraries. Sequencing of virome libraries was conducted on an illumina NovaSeq 6000 (PE150). After sequencing, raw reads were first filtered with an in-house perl script to obtain clean reads without adapter sequences. At the same time, Q20, Q30 and GC content of the clean reads were calculated. De novo assembly was performed with Trinity to obtain Trinity.fa sequences. The Trinity.fa sequences were compared in GenBank Virus RefSeq, NR, CDD and in-house norovirus databases separately to obtain all virus sequences.Lastly, all of the virus sequences were annotated at all classification levels.

## Results

### Real-time RT-PCR detection

From 2015 to 2019, 1,181 samples from sporadic AGE cases, 281 samples from outbreak AGE cases, and six sewage samples were collected in Sichuan. A total of 242 samples from sporadic cases were tested norovirus-positive (240 GII and two GI) while 149 samples from outbreak AGE cases were tested norovirus-positive (138 GII and 11 GI). All six sewage samples tested positive for GI and GII norovirus.

### Molecular characteristics of norovirus in sporadic cases of AGE

**Fig. 1:**
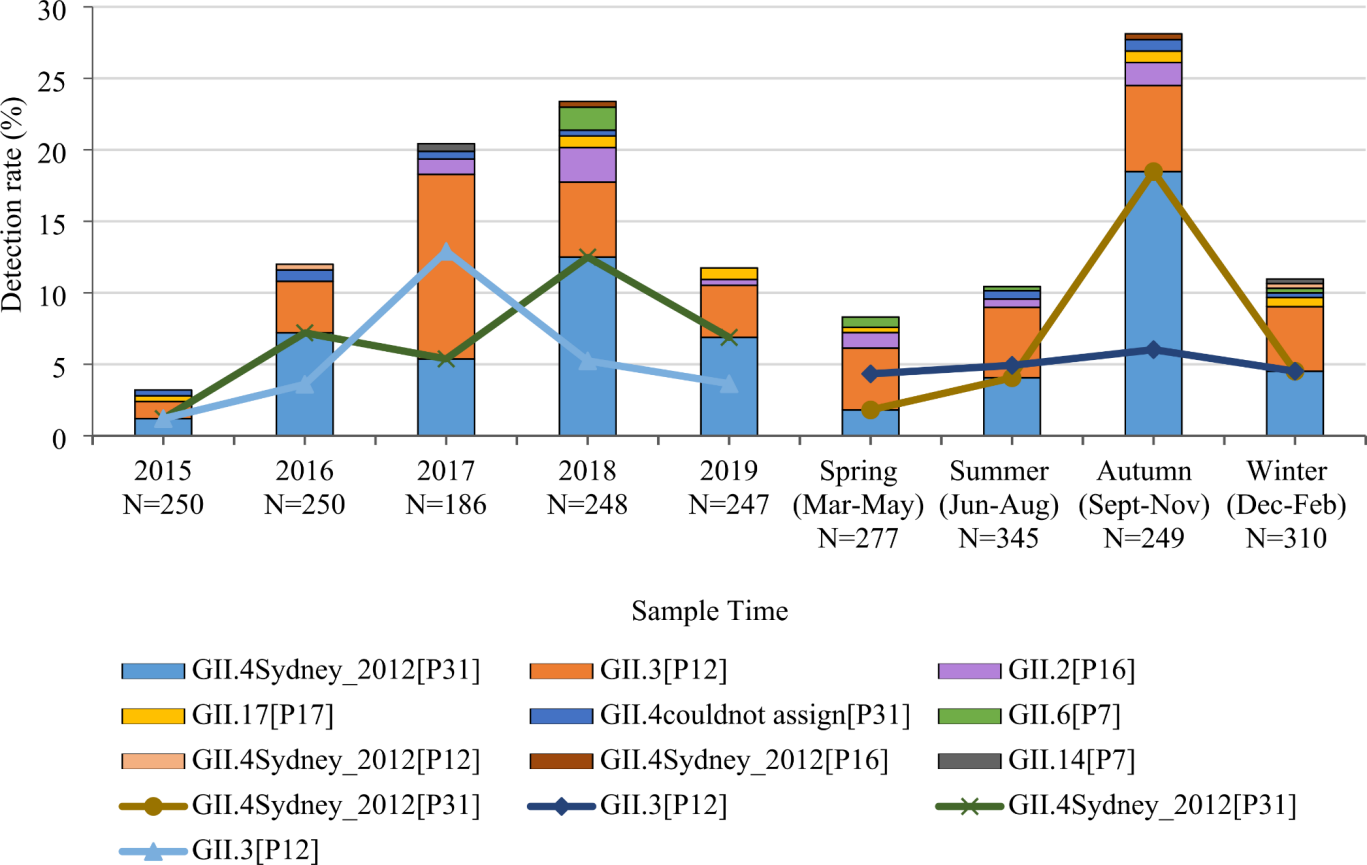
Annual and seasonal distribution of norovirus genotypes in sporadic cases of AGE in Sichuan, 2015–2019

**Fig. 2:**
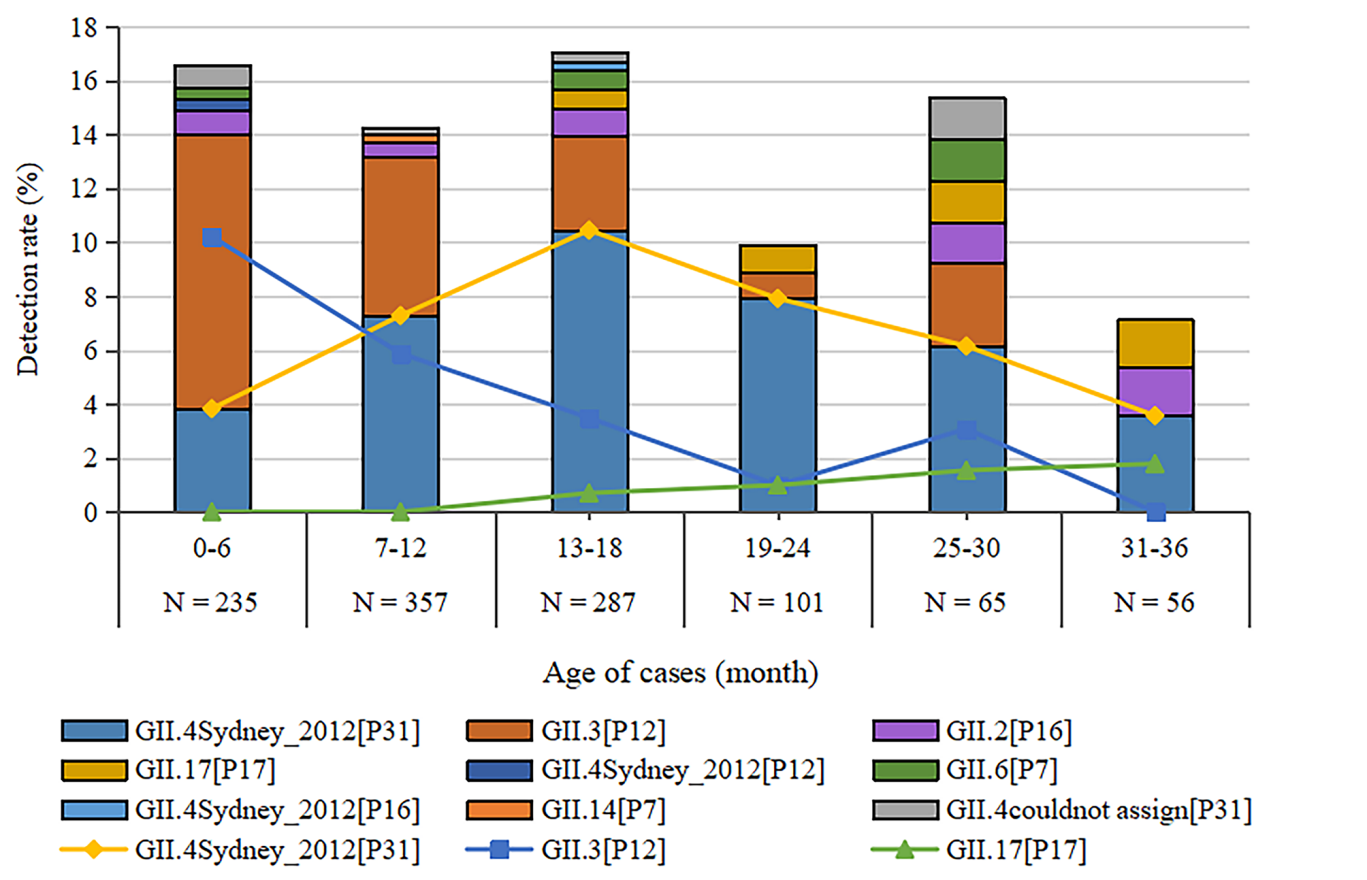
Norovirus genotypes stratified by age in sporadic cases of AGE in Sichuan, 2015–2019

Of the 242 norovirus-positive samples from sporadic cases of AGE, 163 were successfully sequenced and nine genotypes were identified. GII.4 Sydney 2012 [P31] and GII.3 [P12] were the predominant genotypes (Table 1). The total number of genotypes detected each year varied, i.e., 2015 (four genotypes), 2016 (four genotypes), 2017 (five genotypes), 2018 (eight genotypes), and 2019 (four genotypes). GII.4 Sydney 2012[P31] was the predominant genotype each year, except for GII.3 [P12] genotype in 2017. GII.2 [P16] was first detected in 2017 in Sichuan. After being detected in 2015, GII.17 [P17] was not detected again until 2018 (Fig. 1). Predominant genotypes were varied from season and age. Specifically, GII.4 Sydney 2012[P31] was predominant in autumn (September to November) while GII.3 [P12] was predominant in spring (March to May) and summer (June to August) (Fig. 1). GII.3 [P12] was predominant in children 0–6 months of age, while GII.4 Sydney 2012 [P31] was predominant in children older than 6 months. GII.17 [P17] was not detected in children 0–12 months of age, but the detection rate gradually increased with age (Fig. 2).

**Table 1:** **Genotypes of norovirus in sporadic cases of AGE in Sichuan, 2015**–**2019**

Genotype	No.
GII.4 Sydney 2012[P31]	79
GII.3[P12]	58
GII.2[P16]	9
GII.17[P17]	5
GII.6[P7]	4
GII.4 Sydney 2012[P12]	1
GII.4 Sydney 2012[P16]	1
GII.14[P7]	1
GII.4 could not assign[P31]	5
Total	163

### Molecular characteristics of norovirus in outbreak cases of AGE

**Fig. 3:**
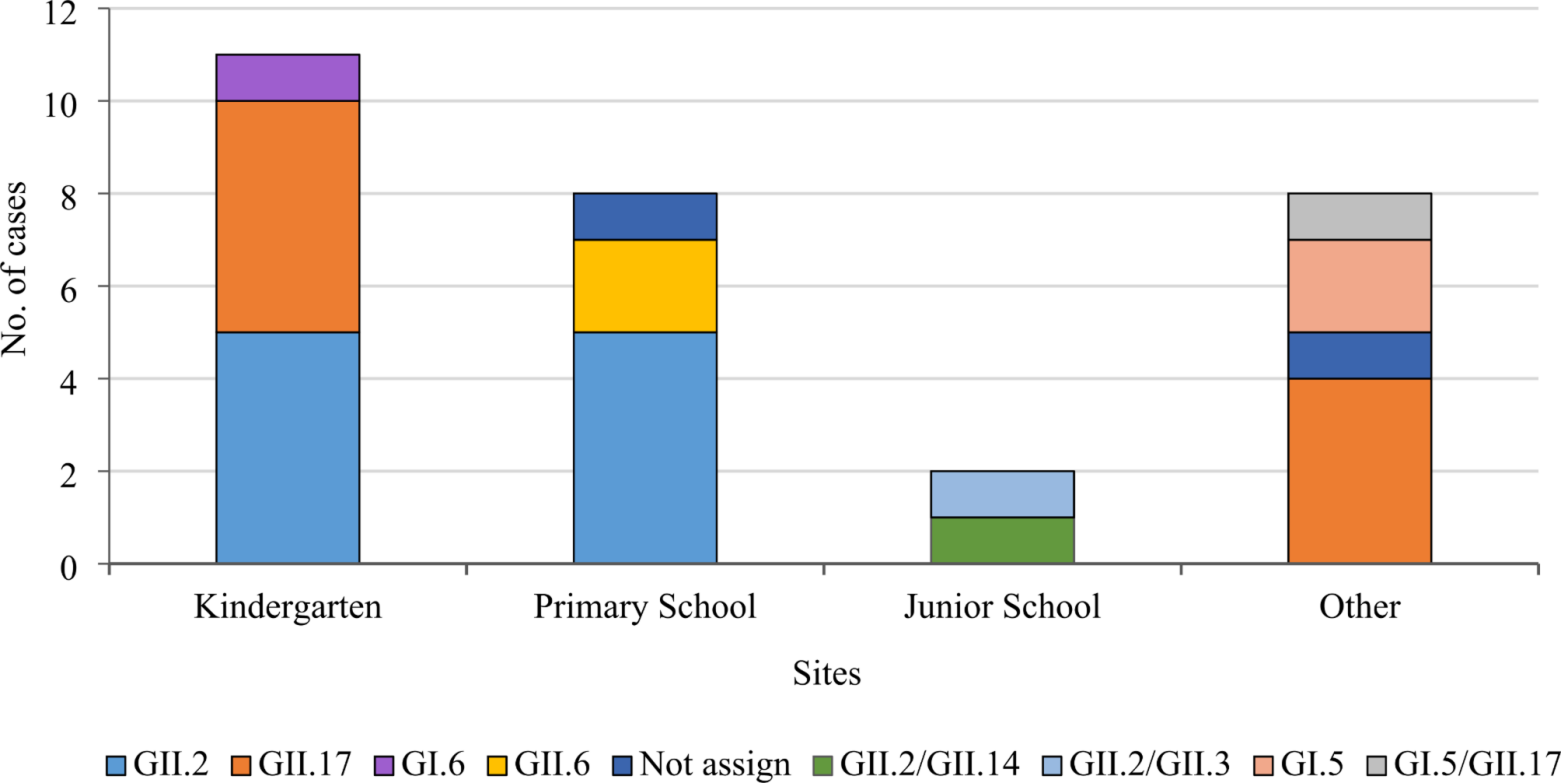
Annual and seasonal distribution of norovirus genotypes in outbreak cases of AGE in Sichuan, 2017–2019

**Fig. 4:**
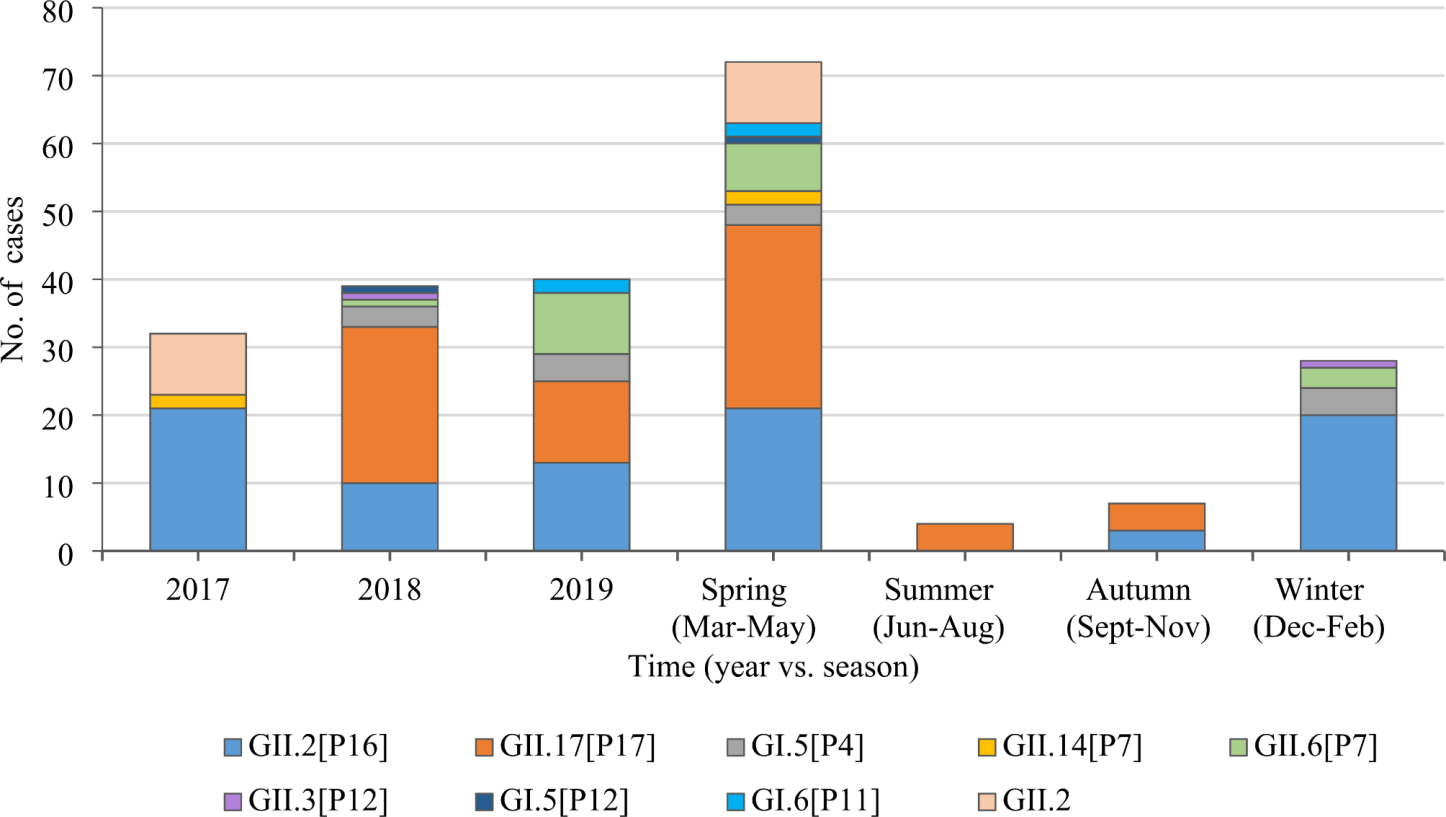
Distribution of norovirus genotypes in different outbreak settings in Sichuan, 2017–2019

Of the 149 norovirus-positive outbreak samples, 102 were successfully sequenced with eight genotypes identified (Table 2). GII.2 [P16] was the primary genotype detected in 2017 while the detection rate of GII.17 [P17] increased each year. Of all 29 norovirus outbreaks, 18 occurred in the spring (March to May) and six in the winter (December to February). Outbreaks during spring were caused by GII.2 [P16] and GII.17 [P17] while outbreaks in the winter were mainly associated with GII.17 [P17] (Fig. 3). Norovirus outbreaks occurred in kindergartens, primary and middle schools, hospitals, companies, building sites, and communities. GII.2 was primarily detected in kindergartens and in primary and middle schools, while GII.17 was largely detected in hospitals, companies, building sites, and communities (Fig. 4).

**Table 2:** **Genotypes of norovirus in outbreak cases of AGE in Sichuan, 2017**–**2019**

Genotypes	No. of outbreaks	No. of cases
GII.2[P16]	8	44
GII.17[P17]	7	35
GI.5[P4]	2	7
GII.14[P7]	1^a^	2
GII.6[P7]	4	10
GII.3[P12]	1^b^	1
GI.5[P12]	1^c^	1
GI.6[P11]	1	2
GII.2[Not assign]	5	9
Not assign	2	38
Total	29	149

Note: a, b, c indicated three outbreaks caused by multiple norovirus genotypes

### Molecular characteristics of norovirus in sewage

Through amplicon sequencing, 1,723,427 raw reads were obtained and subsequently clustered into 7,500 sequences. After blasting in NCBI and in-house norovirus database, 7,137 clustered sequences were annotated. Of the 15 norovirus genotypes identified, eight (GI.1-GI.4, GI.7, GII.12, GII.13, and GII.15) were not detected in sporadic or outbreak cases of AGE in the same period (Table 3).

**Table 3:** Norovirus genotypes in sewage (amplicon sequencing)

Genotypes (GI)	Relative abundance (%)	Genotypes (GII)	Relative abundance (%)
GI.1*	19.24	GII.2	3.58
GI.2*	11.25	GII.3	23.19
GI.3*	2.66	GII.4	21.99
GI.4*	1.69	GII.6	0.05
GI.5	9.99	GII.12*	0.20
GI.6	5.51	GII.13*	0.35
GI.7*	0.03	GII.15*	0.03
		GII.17	0.24

Note: * indicates genotypes that were not found in sporadic cases or outbreak cases of norovirus-related AGE in the same period

Through virome sequencing, 65,859,634 raw reads and 557,242 Trinity.fa sequences were obtained. Subsequently, 805 viral sequences were identified after blasting in the GenBank Virus RefSeq, NR, CDD, and in-house Sichuan norovirus databases. Among the 805 viral sequences, 17 of the sequences were *Caliciviridae*. These 17 *Caliciviridae* sequences were further identified as *Vesivirus* (five), *Sapovirus* (seven), *Norovirus* (four), and *Recovirus* (one). The four *Norovirus* sequences were identified as GIV, GII.3, GII, and GII.15. Besides *Calicivirus*, *Tobamovirus, Mamastrovirus, Picobirnavirus, Ipomovirus, Levivirus, Potexvirus, Rotavirus*, and *Carlavirus* were the top species at genus level in sewage.

## Discussion

Norovirus is highly diverse and multiple genotypes are co-circulating worldwide to cause AGE. However, the distribution of norovirus genotypes varies by region, age, season, and setting. In this study, we investigated the molecular characteristics of norovirus circulating in Sichuan by analyzing surveillance data from multiple years of norovirus positive sporadic and outbreak specimens. A total of 11 distinct genotypes were detected. Among them, GII.4 and GII.3 were the most common genotypes in sporadic infections while GII.2 and GII.17 were most common in outbreak infections. These findings revealed that the dominant genotypes were different between sporadic cases and outbreak cases. In addition, these data suggested that vaccines for preventing norovirus gastroenteritis should protect against these four genotypes at minimum to induce broad protective immunity.

GII.4 Sydney 2012 norovirus has been circulating globally since 2012. From 2014 to 2015, non-GII.4 strains, such as GII.17 and GII.2, were prevalent in Asian regions. However, from 2015 to 2019, GII.4 Sydney 2012 remained as the predominant genotype in Sichuan. As shown in our previous report, norovirus had the highest detection rate in 2018 [10]. There may be three reasons that explain this trend. First, the number of norovirus genotypes was the highest in 2018. The increase in gene diversity may cause an increase in incidence. Second, GII.4 Sydney 2012 was the predominant genotype in 2018, indicating that this virus may be the main genotype leading to this increase in incidence. Third, the nucleotide homology of GII.4 norovirus was lower than other genotypes circulating in the same time period. GII.4 strains collected in 2018 had the lowest nucleotide homology, indicating that GII.4 was more variable in 2018, which may have also led to an increase in incidence. Our previous study showed that autumn was the predominant season of norovirus gastroenteritis in Sichuan [10]. In the current study, GII.4 norovirus was responsible for the majority of norovirus infections in the autumn, further confirming that it continues to be the predominant genotype in Sichuan.

Our previous study indicated that norovirus was more likely to infect children 0–12 months of age than rotavirus in Sichuan [10]. The current study further demonstrated that children 0–6 months of age were primarily infected by GII.3 while children older than 6 months were mainly infected by GII.4. Similar results were reported from Novosibirsk, Russia where GII.3-positive children were younger than GII.4-positive children [11]. These findings revealed that the median age of susceptible children was different depending on the norovirus genotype. In conclusion, in addition to GII.4, GII.3 should be considered for the development of vaccines for infants and young children.

In late 2014, an emergent variant of a previously rare norovirus genotype, GII.17, became dominated in Asian regions, including mainland China [12–14]. GII.17 was detected in 2015 in sporadic AGE cases but not subsequently identified in 2016 or 2017 in Sichuan. The sequences of all strains detected from 2015 to 2019 were highly homologous. However, GII.17 was the second etiology responsible for norovirus outbreaks. It was reported that GII.17 was most prevalent in adult patients and these patients were significantly older than those patients infected with GII.4 [13]. Our current study also showed that GII.17 was not detected in children 0–12 months of age while the detection rate increased with age in children older than 13 months. In addition, five out of seven GII.17 outbreaks occurred in hospitals, companies, building sites and communities. These locations were typically occupied by adults, supporting that GII.17 was a genotype that primarily infects adults.

In the winter of 2016–2017, GII.2 [P16] replaced GII.17 as the predominate genotype in China and accounted for 81.2% of norovirus outbreaks from 2016 to 2018 [15–18]. Interestingly, GII.2 [P16] was also the predominant genotype detected in sewage in Sichuan. However, GII.2 [P16] was rarely detected in sporadic norovirus cases. It was reported that GII.2 [P16] resulted in a higher viral load than GII.17 and GII.4 [19]. In the current study, none of the genotypes detected were found to have a higher viral load.

Surveillance of norovirus was largely based on clinical cases of AGE in sentinel hospitals in China and there was only one sentinel hospital in Sichuan. This hospital-based surveillance system was not adequate to fully evaluate the epidemiology of norovirus. First, this system primarily covered populations under five years of age. Second, cases included in this surveillance system were patients with severe AGE symptoms who sought medical care. Mild or asymptomatic norovirus infections remained unknown. Also, detection rates of some norovirus genotypes were low in clinical cases. Therefore, it is crucial to improve the current surveillance system to obtain comprehensive epidemiological data on norovirus.

Environmental sewage surveillance systems have been established in some developed countries to serve as an effective supplement to the current clinical case surveillance [20–23]. These new systems could yield epidemiological data of norovirus on the view of whole population. And a variety of norovirus genotypes, including genotypes not commonly found in clinical cases, were detected by sewage surveillance. Besides, establishing of sewage surveillance could not only provide information about norovirus disease but also information about other contagious diseases, especially some emerging or re-merging diseases. This surveillance system will likely be more effective and more economic than case surveillance after taking full advantage of these data. Thus, this study set up environmental sewage surveillance system and described the molecular characteristics of norovirus in sewage in Sichuan. Using amplicon sequencing, 15 norovirus genotypes were identified, of which eight genotypes had not been detected in clinical cases in the same period. Notably, GII.3 was secondary to GII.4 in sporadic AGE cases while the relative abundance of GII.3 in sewage had exceeded GII.4 in Sichuan. In China from 2015 to 2016, the number of GII.3 norovirus outbreaks increased compared with previous years [24]. In Beijing in 2017, GII.3 was found to have a higher detection rate than GII.4 in hospitalized children with AGE [25]. To determine whether the detection rate of GII.3 would exceed that of GII.4 in clinical cases, long-time surveillance is need, which should be a focus of future monitoring work. A slight majority (50.37%) of norovirus sequences found in sewage were typed as genogroup I. However, in a five-year period, only two sporadic norovirus cases were typed as GI. This may suggest that the current surveillance system was not sensitive enough to detect GI infections or GI noroviruses result more frequently in asymptomatic infections.

## Conclusion

Relying on data collected from sporadic and outbreak AGE cases and environment sewage, this study established a comprehensive surveillance system and enhanced the ability to monitor the molecular characteristics of norovirus circulating in Sichuan. GII.2, GII.3, GII.4, and GII.17 were the predominant genotypes. However, these predominant genotypes varied from population, age, season, and setting. Additionally, these data helped to clarify the epidemiological characteristics of norovirus and the development of vaccines, as well as future interventions for norovirus gastroenteritis. Furthermore, the complete genome of a selection of each norovirus genotype should be sequenced, thereby providing a greater understanding of the molecular characteristics and evolutionary mechanisms of norovirus.

## Data Availability

The original contributions presented in the study are included in the article. The datasets used and/or analyzed during the current study are available from the corresponding author on reasonable request.

## Abbreviations

*AGE*: Acute gastroenteritis.
*RdRp*: RNA-dependent RNA polymerase.
*CDC*: Center for disease control and prevention.
*RT-PCR*: Reverse transcription polymerase chain reaction.
